# Molecular Mechanisms of Bone Metastasis: Which Targets Came from the Bench to the Bedside?

**DOI:** 10.3390/ijms17091415

**Published:** 2016-08-27

**Authors:** Sandra Casimiro, Arlindo R. Ferreira, André Mansinho, Irina Alho, Luis Costa

**Affiliations:** 1 Instituto de Medicina Molecular, Faculdade de Medicina, Universidade de Lisboa, 1649-028 Lisbon, Portugal; scasimiro@medicina.ulisboa.pt (S.C.); ajrsferreira@medicina.ulisboa.pt (A.R.F.); ialho@medicina.ulisboa.pt (I.A.); 2 Oncology Division, Hospital de Santa Maria, Centro Hospitalar Lisboa Norte, 1649-028 Lisbon, Portugal; andre.mansinho@chln.min-saude.pt

**Keywords:** bone metastases, vicious cycle of bone metastases, osteotropic factors, pre-metastatic niche, bone-targeted agents, bisphosphonates, denosumab, radium-223

## Abstract

Bone metastases ultimately result from a complex interaction between cancer cells and bone microenvironment. However, prior to the colonization of the bone, cancer cells must succeed through a series of steps that will allow them to detach from the primary tumor, enter into circulation, recognize and adhere to specific endothelium, and overcome dormancy. We now know that as important as the metastatic cascade, tumor cells prime the secondary organ microenvironment prior to their arrival, reflecting the existence of specific metastasis-initiating cells in the primary tumor and circulating osteotropic factors. The deep comprehension of the molecular mechanisms of bone metastases may allow the future development of specific anti-tumoral therapies, but so far the approved and effective therapies for bone metastatic disease are mostly based in bone-targeted agents, like bisphosphonates, denosumab and, for prostate cancer, radium-223. Bisphosphonates and denosumab have proven to be effective in blocking bone resorption and decreasing morbidity; furthermore, in the adjuvant setting, these agents can decrease bone relapse after breast cancer surgery in postmenopausal women. In this review, we will present and discuss some examples of applied knowledge from the bench to the bed side in the field of bone metastasis.

## 1. Introduction

Bone is the most frequent affected organ by metastatic disease in the two most frequent cancers in the Western world: breast cancer and prostate cancer. The special attraction of breast cancer cells to bone was recognized by Paget in the nineteen century and is still considered one of the fundamental discoveries in cancer research [1]: “the seed and soil hypothesis”. Until now, many other investigators have been devoting their time and effort to unravel at the molecular level the organotropic attraction of cancer cells to different organs [2,3,4].

The pathophysiology of bone metastases is mostly explained by the theory of the vicious cycle proposed by Mundy and Guise [5]. According to this theory, cancer cells resident in bone can cause bone destruction because they are capable to stimulate osteoclast activity and, in return, cancer cells will receive positive feed-back from humoral factors released by the bone microenvironment during bone destruction and altered remodeling. Indeed, it is widely accepted that the bone microenvironment is crucial to the success of cancer cells in bone [6].

Interestingly, since the discovery of the role of bisphosphonates (BPs) as bone-targeted agents (BTAs) that inhibit osteoclast activity, the treatment of bone metastasis is considered as the best example that combining strategies (anti-cancer agents with microenvironment targeting agents) can lead to a better outcome in patients affected by bone metastasis. Indeed, several large phase 3 trials have proven the benefit of BPs and of denosumab (a monoclonal antibody that neutralizes receptor activator of nuclear factor kappa-B ligand (RANKL)) by decreasing the morbidity associated with bone metastasis. These BTAs can decrease the incidence of bone complications such as pathologic fractures, spinal cord compression, need of radiation to control pain or to prevent a fracture, among others [7,8]. This capacity to decrease the incidence of bone complications is observed across many tumor types with the most potent BTAs (denosumab and zoledronic acid (ZA)), thus suggesting that the vicious cycle theory is applicable in most solid tumor types once cancer cells reach the bone and start to interact with bone microenvironment.

Furthermore, in the adjuvant setting, BPs and denosumab showed to impact favorably the incidence of bone metastases in postmenopausal women after surgery for breast cancer. This is one of the first examples of a host-directed therapy with impact in cancer relapse [9,10,11].

Therefore, the expectations are real when we hope to improve our knowledge about the mechanisms of bone metastases to achieve better clinical outcomes. In this review, we aimed to reveal and to discuss some examples of applied knowledge from the bench to the bed side in the field of bone metastasis.

## 2. Pathophysiology of Bone Metastases

In 1889, Paget observed in a series of autopsies that cancer cells do not grow randomly in secondary locations, but instead exhibit specific tropism for certain organs, and proposed the visionary “seed and soil” theory of metastasis formation [12]. We now know that the metastatic cascade is a complex step by step process that includes invasion, intravasation, survival in the circulation, extravasation, and seeding at a distant site or colonization [13]. Each one of these steps depends on specific molecular and biological features of tumor cells but also on the interaction with different microenvironments. Colonization of the bone, the final step towards the growth of a bone metastasis, ultimately results from the unique ability of cancer cells to adapt and grow in a congenial microenvironment that they will further manipulate to their own profit, in a so-called “vicious cycle of bone metastases” [14].

### 2.1. The “Vicious Cycle of Bone Metastases”

**Figure 1:**
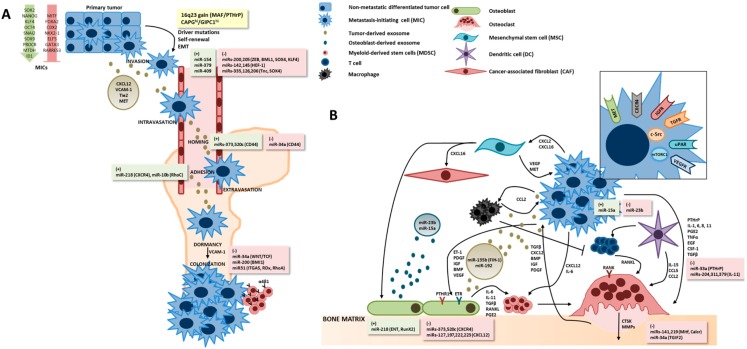
Molecular players of the metastasis formation cascade in the bone (**A**) and “bone vicious cycle” (**B**). (**A**) Metastasis-initiating cells (MICs) are a small fraction of long-term self-renewing tumor-initiating cells, characterized by specific driver mutations and high cellular plasticity [22]. Osteotropic events, like the 16q23 gain or macrophage-capping protein (CAPG)/ PDZ domain-containing protein GIPC1 (GIPC1) overexpression, will occur at early stages of tumor development [23,24]. Exosomes released from the primary tumor can facilitate the establishment of the pre-metastatic niche by transporting cytokines or being involved in gene transfer [22]. Upon the dormancy phase, vascular cell adhesion molecule 1 (VCAM-1) promotes osteolytic expansion of indolent bone micrometastases by engaging α4β1-positive osteoclast progenitors [25]; (**B**) the “vicious-cycle of bone metastases” results from the complex interaction between tumor cells, bone forming osteoblasts, bone resorbing osteoclasts, and a variety of cells from the bone microenvironment and immune system, like cancer-associated fibroblasts (CAFs), mesenchymal stem cells (MSCs), dendritic cells (DCs), T cells and macrophages [26]. Osteoblasts are activated by tumor-derived parathyroid hormone-related protein (PTHrP), leading to increased production of receptor activator of nuclear factor kappa-B ligand (RANKL). RANKL binds to receptor activator of nuclear factor kappa-B (RANK) expressed on hematopoietic osteoclast precursors, leading to osteoclastogenesis and bone resorption. Bone matrix-stored minerals and growth factors are released and activated, further feeding the tumor cell growth. In osteoblastic bone metastases, like in prostate cancer, osteoblasts activity is stimulated by several growth factors like basic fibroblast growth factor (bFGF), bone morphogenetic proteins (BMPs), endothelin 1 (ET-1), transforming growth factor beta (TGFβ) and insulin-like growth factor 1 (IGF-1), and deposition of disorganized new bone matrix is exacerbated [27]. miRNAs can act as master regulators of gene expression, having a positive (+) or negative (−) effect on specific genes that will control multiple aspects of bone metastasis formation [28]. BMI1, polycomb complex protein; CCL-2, Ccl2 chemokine (C–C motif) ligand 2; CD44, cell Surface Glycoprotein CD44; CTSK, cathepsin K; CSF-1, colony stimulating factor 1; CXCL12, C–X–C motif chemokine 12; CXCR4, C–X–C chemokine receptor type 4; EGF, epidermal growth factor; EMT, epithelial-mesenchymal transition; ENT, equilibrative nucleoside transporter 1; ETR, endothelin receptor; FIH-1, factor Inhibiting HIF1; HEF-1, human enhancer of filamentation 1; IGFR, insulin-like growth factor 1 (IGF-1) receptor; IL, interleukin; KLF4, kruppel-like factor 4; ITGA5, integrin Subunit Alpha 5; MAF, V-Maf avian musculoaponeurotic fibrosarcoma oncogene homolog; MET, hepatocyte growth factor receptor; MMP, matrix metalloproteinases; mTORC1, mammalian target of rapamycin complex 1; PDGF, platelet-derived growth factor; PGE2, prostaglandin E2; PTH1R, parathyroid hormone 1 receptor; RDx, radixin; SOX4, transcription factor SOX-4; TCF, transcription factor family; TGIF2, TGF beta induced factor homeobox 2; TGFR, transforming growth factor beta receptor II; uPAR, urokinase receptor; VEGF, vascular endothelial growth factor; VEGFR, vascular endothelial growth factor receptor; WNT, wingless-related integration site proteins; ZEB, zinc finger E-box-binding homeobox 1.

Since the initial steps of metastasis formation may occur early during tumor development and therefore escape detection (Figure 1A), the therapeutic focus has been on the colonization step, which constitutes the critical transition between micrometastases and macrometastasis [15]. The “vicious cycle of bone metastases” has been deeply dissected, and our knowledge on the molecular players and mechanisms has evolved substantially (Figure 1B) [16,17,18]. 

Whereas bone normal physiology depends on a strict equilibrium between bone resorption by osteoclasts and new bone formation by osteoblasts, cancer cells growing at bone disrupt this balance favoring bone resorption. In physiologic bone remodeling monocyte or macrophage precursors differentiate into osteoclasts via colony-stimulating factor-1 (CSF-1), receptor activator of NFκB ligand (RANKL), interleukin 6 (IL-6), interleukin 8 (IL-8) and chemokine (C–C motif) ligand 12 (CCL2) [19,20]. Osteoclast-derived bone resorption leads to the release of osteoblastic factors like transforming growth factor β (TGF-β), bone morphogenetic proteins (BMPs), fibroblast growth factor (FGF), platelet-derived growth factor (PDGF), and insulin-like growth factors (IGFs) trapped into the bone matrix, which then induce osteoblastogenesis derived from stromal mesenchymal stem cells (MSCs). The molecular regulators of this process are the RANKL–RANK–osteoprotegerin (OPG) triad [20,21]. The receptor activator of NFκB (RANK) is a transmembrane receptor expressed on the surface of osteoclast precursor cells, being activated by its ligand RANKL, which is produced by osteocytes, osteoblasts and bone marrow stromal cells. RANK activation by RANKL regulates osteoclast differentiation, activity and survival. RANKL, and subsequent RANK pathway activation, may be blocked by RANKL soluble decoy receptor osteoprotegerin (OPG), which is secreted by mature osteoblasts and stromal cells.

Osteolytic metastases, like in breast cancer, are consequence of increased bone resorption, orchestrated by tumor cells. Tumor-derived osteoclastogenic factors, such as interleukin 1 (IL-1), IL-6, parathyroid hormone-related protein (PTHrP), prostaglandin E2 (PEG2), CSF-1 and tumor necrosis factor-α (TNFα) drive an increase in bone resorption. RANKL expression is up-regulated by increased PTHrP and interleukin 11 (IL-11) in the bone microenvironment, which leads also to a down-regulation of OPG and activated osteoclastogenesis. As a consequence, mitogenic factors are released from the bone matrix, such as TGF-β, IGFs, FGFs, PDGF and Ca^2+^, further feeding the “vicious cycle” of tumor growth and bone resorption.

Osteoblastic bone metastases occur mostly in prostate cancer. In this case, together with the increase in osteoclastogenesis, osteoblasts are also activated by prostate cancer cells and the tumor microenvironment leading to the accumulation of new formed bone. Prostate cancer cells growing at bone express endothelin-1 (ET-1) that stimulates osteoblasts via the endothelin A receptor (ETR), activating Wnt signaling [29]. Tumor-derived proteases like matrix metalloproteinases (MMPs), prostate-specific antigen (PSA), or urokinase-type plasminogen activator (uPA) contribute to the release of osteoblastic factors from the extracellular matrix, including TGF-β and IGF-I [30]. Increased tumor-induced osteoblast activity further increases RANKL and decreases Ca^2+^, leading to parathyroid hormone (PTH) release, promoting osteoclast activity.

Despite our major efforts in dissecting and targeting the “vicious cycle of bone metastases”, the success has been limited, with exception of bone-targeted therapies, like bisphosphonates (BPs), denosumab or Ra-233, meaning that the complexity of the system is somehow impairing an effective role of anti-tumoral agents. Therefore, efforts have been made on the comprehensive analysis of the previous steps of cancer progression towards bone relapse. Several studies demonstrate that miRNAs can control multiple aspects of bone metastasis formation, including cancer cell escape from the primary tumor site, cancer cell dissemination to bone and invasion of the bone marrow, as well as secondary outgrowth and tumor–stroma cell interactions [28]. Moreover, the concepts of the existence of metastasis-initiating cells (MICs) [31] and of a “pre-metastatic niche” [26,31,32,33] are being intensively explored. Next, we will highlight some important findings that help to fill the gaps in our knowledge of the pathophysiology of bone metastases.

### 2.2. Metastasis-Initiating Cells, Chemoattraction to Bone and the “Pre-Metastatic Niche”

MICs are defined as unique populations of cells that benefit from unique traits that may originate in the primary tumor but change during metastasis formation cascade and colonization. Upon extravasation at bone marrow, MICs adopt the bone marrow pre-existing endosteal niche, which normally functions to support hematopoietic stem cells (HSCs), to enhance their survival in a foreign microenvironment. Importantly, MICs at primary tumor or disseminated tumor cells (DTCs) will also induce a so-called “pre-metastatic niche”, altering the expression of extracellular matrix components and mobilizing bone marrow progenitor cells, to create a beneficial microenvironment for seeding and growth. The notion that secondary bone microenvironment can be prepared for future metastatic growth before the arrival of tumor cells is probably in the base of the successful trials on the adjuvant use of bone-targeted therapies. Amongst the distinct traits that MICs may have it was recently shown that the 16q23 gain is selectively associated with bone metastasis risk in early-stage breast cancer and that the transcription factor V-Maf avian musculoaponeurotic fibrosarcoma oncogene homolog (MAF) encoded within this region acts as a mediator of metastasis to bone by regulating the expression of the osteotropic factor PTHrP [23]. This reinforces the idea that MICs will prime the “pre-metastatic niche” prior to their arrival. In addition, high expression of macrophage-capping protein (CAPG) and PDZ domain-containing protein GIPC1 (GIPC1) in primary breast tumors was shown to predict disease outcomes, being these patients more likely to develop first distant recurrence in bone, although their role in osteotropism is yet unknown [24]. These proteins were also shown to be biomarkers of benefit from ZA and may facilitate patient selection for adjuvant BPs treatment.

Exosomes derived from MICs may also be implicated in priming bone microenvironment. Exosomes released from hormone-refractory prostate cancer cells have been shown to facilitate mouse pre-osteoblast differentiation and to increase MSCs differentiation into myofibroblasts that promote tumor growth [34,35].

The tropism that cancer cells exhibit towards certain organs depends in part on their chemokine/chemokine receptors expression profile. The secretion of C–X–C motif chemokine 12 (CXCL12), also known as stromal-derived factor 1 (SDF-1), by stromal cells in the bone marrow is known to attract osteotropic cancer cells via stimulation of the C–X–C chemokine receptor type 4 (CXCR4) receptor that is up regulated by many tumors. Therefore, the CXCL12/CXCR4 axis contributes to the establishment of an appropriate microenvironment in the bone, or a “pre-metastatic niche”, for osteotropic cancers like breast, and prostate [36,37]. This was first demonstrated in breast cancer cells, where signaling through CXCR4 was shown to mediate actin polymerization and pseudopodia formation, and induce chemotactic and invasive responses [38]. CXCR4 was then identified as part of a gene signature of bone metastases, and its overexpression was sufficient to increase bone metastases in vivo [39]. Moreover, blocking CXCR4 expression at the mRNA level decreased breast cancer cell invasion in in vitro assays and inhibited metastasis in an animal model [40]. In prostate cancer, CXCR4 expression is associated with higher tumor grade, and the use of a neutralizing antibody to CXCR4 limited the extent of bone metastases and growth of intraosseous prostate cancer cells after intratibial injections [41,42]. Posteriorly, CXCL12 signaling through CXCR4 was shown to trigger the adhesion of prostate cancer cells to bone marrow endothelial cells by activating αvβ3 integrins [43]. In addition, using a prostate cancer model, CXCR4-dependent migration was shown to increase matrix metalloproteinase-9 (MMP-9) expression and decrease TIMP metallopeptidase inhibitor 2 (TIMP2) expression, contributing to a more invasive phenotype [44,45].

The disruption of the “pre-metastatic niche” or the overcome of “pre-metastatic niche”-induced growth restrictions will be responsible for the end of the dormancy phase. Using a mouse model of metastasis it was shown that human prostate cancer cells directly compete with HSCs for occupancy of the mouse HSC niche, and that the niche size was correlated with dissemination [46]. Once in the niche, tumor cells reduced HSC numbers by driving their terminal differentiation. Moreover, it has been shown that HSCs derived from mice bearing subcutaneous PC3 tumors can induce the differentiation of HSCs into osteoclasts, via IL-6 [47]. Additionally, quiescence and proliferation switch is also regulated by tyrosine-protein kinase receptor UFO (AXL) or tyrosine-protein kinase receptor TYRO3 precursor (TYRO3) signaling, respectively, as AXL and TYRO3 protein kinases compete for the growth arrest-specific 6 (GAS6) ligand secreted by osteoblasts [48]. In a breast cancer bone metastasis dormancy model, it was shown that aberrant expression of vascular cell adhesion molecule 1 (VCAM-1) promoted the transition from indolent micrometastases to macrometastases [25]. It was further demonstrated that VCAM-1 recruits monocytic osteoclast progenitors and elevates local osteoclast activity by interacting with the cognate receptor integrin α4β1. In another study, it was shown that breast cancer MICs located in the bone predominantly reside in a niche that exhibits features of osteogenesis, and that that niche interactions were mediated by heterotypic adherens junctions involving cancer-derived E-cadherin and osteogenic N-cadherin [49]. Moreover, the activation of protein kinase B (AKT)/mammalian target of rapamycin complex 1 (mTORC1) signaling within luminal breast cancer cells, which were in direct contact with osteoblasts at the “pre-metastatic niche”, promoted osteoblast-induced breast cancer proliferation and the progression to macrometastases, leading to a transition from a pre-osteolytic phase to an osteolytic phase. 

The hypothesis that bone metastases are primed at early stages of tumor progression, and that the “pre-metastatic niche” is crucial for their development offers a unique opportunity for therapeutic development. Targeting early molecular determinants, exosome release and/or content, or the “pre-metastatic niche” may be useful for the prevention of bone metastases. Therefore, efforts have been made to identify early molecular determinants that could predict bone relapse.

### 2.3. Genomic Signatures of Bone Metastases

Gene expression profiles have been used for more than one decade to stratify patients with breast cancer according to the risk of relapse or benefit from therapy. This is the case of the prognostic *van’t Veer’s* 70-gene signature [50] (Mammaprint), validated in several retrospective studies [51,52,53,54,55,56,57]; or the 21-gene recurrence score (Oncotype Dx) [58], also validated through retrospective or prospective analyses [59,60,61,62]. However, none of these gene profiles discriminates patients according to their risk of bone-specific, or in fact other organ-specific, relapse.

Breast cancer organ-specific tropism has been a hot topic in research for many years and major insights are mostly derived from studies with animal models. The seminal studies from Massague’s group analyzed the genomic profiling of organ-tropic metastatic variants derived from human breast cancer cell lines injected in immune-compromised mice, and used comparative genomic analyzes in clinical specimens to identify bone, lung and brain metastases-specific gene expression signatures [4,39,63]. However, none of these signatures have proven to be predictive of organ-specific risk of relapse when analyzing primary tumor material. 

A subsequent study showed that *Kang’s* bone-metastasis signature hierarchical clustering of a mixed cohort of primary breast tumors was unable to discriminate those tumors that gave rise to bone metastasis vs. those that did not [64]. However, a distinction was made between primary breast carcinomas that preferentially metastasized to bone from those that preferentially metastasized elsewhere, suggesting that the enrichment of the gene signature could allow the prediction of bone metastases in primary breast cancers. 

*Kang’s* bone-metastasis gene signature included membrane or secretory products that may affect the host environment to favor metastasis, namely the chemokine receptor *CXCR4*; the angiogenesis factors *FGF*-*5* and connective tissue growth factor (*CTGF*); the activator of osteoclast differentiation *IL*-*11*; the matrix metalloproteinase/collagenase *MMP-1*; the disintegrin and metalloproteinase with thrombospondin motifs 1 (*ADAMTS1*); and the adhesion molecule osteopontin (*OPN*) [39]. Importantly, genes in this set were not part of *van’t Veer’s* signature, and genes in *van’t Veer’s* signature were not overexpressed in bone-seeking clones, although the parental line carries the poor-prognosis signature. These genes will probably be activated at later stages of cancer dissemination, and will be responsible for the adaptation of the cancer cells to the foreign microenvironment, either promoting seeding or colonization (e.g., *OPN*, and *CXCR4*) rather than dictating their attraction to a certain organ. An exception may be the expression of *CXCR4*, involved in the chemo attraction of cancer cells to CXCL12-rich environments, but not exclusively bone (e.g., brain also). In fact, the experimental model used for the selection of organ-specific clones, like the intra-cardiac injections for bone metastases, mostly mimics the latter steps of metastatic process, thereby possibly contributing to the identification of “late-stage” genes. We analyzed the expression of the bone metastasis gene signature in pure cancer cell populations obtained from bone metastases from patients with different types of solid tumors, including breast cancer [18]. All the analyzed genes were overexpressed in the tumor cells, and no differences were observed between gene expression in bone metastases from breast cancer or from other solid tumors.

Other studies have tried to get insights into the molecular determinants of bone metastases by combining in silico and in vitro or in vivo data. Despite important data and clues found on the biology of bone metastases, no genomic signatures were derived from these efforts. Therefore, attempts have been made to identify gene signatures in the clinical setting rather than in non-clinical models. Smid et al. analyzed 107 primary breast tumors in patients who were all lymph node negative at the time of diagnosis and all had experienced relapse [65]. A total of 69 genes were differentially expressed between patients that relapse to bone vs. those who relapsed elsewhere in the body. In this study, a classifier of 31 genes correctly predicted all tumors relapsing to bone with a specificity of 50% in a validation clinical cohort.

Recently, a gene array analysis of 157 primary breast tumors of patients with known metastatic disease generated a 15-gene signature that could identify 82.4% of the tumors with bone metastasis, 85.2% of the tumors which had bone metastasis as first site of metastasis and 100% of the ones with bone metastasis only (*p* = 9.99 × 10^−09^) [66]. This was also corroborated in an independent cohort of 376 breast cancers (*p* = 4.28 × 10^−10^). Interestingly, *Kang’s* signature showed a sensitivity of 81.5% and specificity of 48.8% in the same training set, being positive in 100% of Luminal A, 90.7% of Luminal B, 33% of HER2-like and 0% of basal-type tumors. The 15-gene signature was predictive in both ER-positive and ER-negative tumors. Importantly, in this signature only three genes related to epithelia-to-mesenchymal transition were overexpressed (*NAT1*, *BBS1* and *PH*-*4*), thus suggesting a role of earlier stages of metastasis formation.

What is striking is that there is little overlap between signatures. A sequential pattern of expression that leads to breast cancer progression and metastasis seems to be recapitulated by the sequential expression of genes in these signatures, that deviate from each other toward distinct functions [67]. Collectively, genes in these signatures probably regulate cellular signaling pathways in primary and secondary breast tumors, like TGF-β, FGF, Janus kinase/signal transducers and activators of transcription (JAK-STAT), nuclear factor kappa-B (NFκB), wingless-related integration site proteins (WNT), and phosphatidylinositol-4,5-bisphosphate 3-kinase (PI3K) pathways in primary tumor, and TGF-β, FGF, NFκB, and PI3K pathways in metastases. 

Thus far, there is no genomic predictor of bone-specific recurrence clinically validated or in trials. Tumor and host heterogeneity, as well as methodological approaches and particularities will probably contribute to the lack of overlapping. Nevertheless, all these efforts have enormously contributed to fill the details of the metastasis formation cascade and the biology behind the “vicious cycle of bone metastases”. This knowledge enabled researchers to test in the clinical arena several mechanistically meaningful targets with heterogeneous results. Over the following sections a revision of the agents that demonstrated a favorable safety and efficacy profile rendering regulatory approval for clinical use will be performed (Table 1 and Table 2). A brief reference to some new forms of targeting these pathways will be also presented (Table 3).

## 3. Validated Targets 

The clinical application of BTAs in current clinical practice is mostly focused on the inhibition of osteoclast activity either by the use of BPs or denosumab. The radiopharmaceutical agent radium-223 (Ra-223) is also considered by many authors as a BTA. Indeed, it has high affinity to bone, particularly to the site of bone metastasis, and does reduce significantly the incidence of bone complications in patients with prostate cancer and bone metastases. However, Ra-223, which is an alpha emitter, is not just a BTA because it also causes direct cancer cell death by radiation-induced double strand breaks. In our perspective, Ra-223 is a mixed therapeutic proposal (BTA and anti-cancer agent). We do not know yet whether Ra-223 can also be effective in targeting osteoblast hyperactivity (like in blastic bone metastases associated with prostate cancer).

We shall describe first the major achievements of BTAs agents in the treatment of bone metastases followed by a brief exposition of their role in the adjuvant setting. 

### 3.1. Bone Targeted Agents in Advanced Disease

Solid tumors with bone metastases are usually complicated by the occurrence of skeletal related events (SREs), a composite endpoint frequently defined in clinical trials by the occurrence of pathologic fractures (incidental or symptomatic), spinal cord compression or radiation/surgery to the bone; in some, hypercalcemia of malignancy is also considered. Symptomatic skeletal events (SSEs) differ from the latter for only including symptomatic pathologic fractures. 

SREs are a frequent clinical event in patients with bone metastases and inflict a significant burden to cancer patients. In prostate cancer, bone metastases occur in up to 70% of cases and, over a two-year period, SREs occur close to half of patients with metastatic castration resistant prostate cancer (mCRPC) with bone metastasis not treated with a BTA [68]. Bone is also the site of first disease recurrence in 30% to 40% of women with breast cancer and approximately 70%–80% will develop bone metastasis throughout the course of the disease, with the complications associated causing significant morbidity and impairing quality of life. In these patients, pain is the most frequent SRE, occurring in up to 75% of patients, pathologic fracture follows with 16%, while the least common is spinal cord compression in 3% [69]. Although more frequent in breast and prostate cancer, bone metastasis can occur in virtually any type of cancer.

When assessing the radiographic appearance of bone metastases, three patterns occur: osteoblastic, osteolytic or mixed, as a function of radiographic density; however, all are associated with an increase in osteoclastic activity, including the osteoblastic metastasis, so multiple osteoclast targeted agents have been studied in this setting [70]. In current clinical practice, only denosumab and BPs are approved for the prevention of SREs in metastatic disease (Table 1). As detailed above, Ra-223 is also considered by some as a BTA.

BPs have high affinity for calcium ions thus attaching to hydroxyapatite binding sites on bone surface, especially those undergoing active resorption. During bone resorption, BPs are internalized by bone-resorbing osteoclasts and inhibit osteoclast function [71]. Nitrogen-containing BPs (alkyl-amino BPs: pamidronate, alendronate, ibandronate; heterocyclic BPs: risendronate, ZA) impair the mevalonate pathway by inhibiting the farnesyl diphosphate synthase (FPP synthase), ultimately preventing prenylation of small GTPase signaling proteins vital for normal cellular function. Non-nitrogen containing BPs (etidronate, clodronate, tiludronate) induce to the formation of deleterious metabolites in osteoclasts. Beyond their effects on osteoclast inhibition, BPs may also have antitumor and/or antiangiogenic effects, but this is a controversial area (see Section 3.4.). Investigations are ongoing to better define the clinically relevant effects of BPs in patients with cancer [72]. Pamidronate and ZA have been approved by both European Medical Agency (EMA) (or local European authorities) and Food and Drug Administration (FDA) for the treatment of skeletal metastases from solid tumors and multiple myeloma (MM). Clodronate is not approved for clinical use in the US but is available in Europe. Ibandronate is also an alternative. ZA is the only BP approved for mCRPC and is also approved for use in patients with other solid tumors.

The first positive clinical trial using BPs in breast cancer goes back to 1987, where 34 normocalcemic breast cancer patients with progressive osteolytic bone metastases were treated with clodronate (1.6 g/day) or placebo for 12 months. Bone pain, extension of bone metastases and formation of new osteolytic foci were reduced by clodronate, and development of severe hypercalcemia was prevented [81]. This was followed by a larger, double-blinded trial in 1993, where 173 patients with bone metastasis due to breast cancer were randomized to oral clodronate (1.6 g/day) or placebo. The proportion of all SREs was substantially reduced (218.6 vs. 304.8 per 100 patient-years; *p* < 0.001). A trend in favor of clodronate was also found in the rates of non-vertebral fracture and need of radiotherapy for bone pain control (particularly spinal pain) [82]. 

Pamidronate, a second generation and oral BP, was investigated on a subsequent randomized study aiming to assess its effects on the morbidity from bone metastases of breast cancer patients and its gastrointestinal tolerability. In the pamidronate group, the occurrence of hypercalcemia of malignancy, symptomatic impending fractures, need for radiotherapy and severe bone pain improved by 65%, 50%, 35% and 30%, respectively [83]. 

Pamidronate was also evaluated in two double-blind, randomized placebo-controlled trials at a dose of 90 mg IV in breast cancer patients who were being treated either with chemotherapy or hormonal therapy. In this study, pamidronate treatment was associated with a significant reductions in SREs and pain. In patients receiving chemotherapy, median time to first SRE was longer in the pamidronate group when compared to placebo (13.1 vs. 7.0 months, *p* = 0.005) and lower proportion of patients with any SRE (43% vs. 56%, *p* = 0.008). In patients receiving hormonal therapy, at 24 cycles, the proportion of patients with an SRE was 56% in the pamidronate group and 67% in those treated with placebo (*p* = 0.027); median time to first SRE was 10 vs. 7 months (*p* = 0.05) for the pamidronate and placebo groups, respectively [84,85].

ZA, a third generation BP, was studied in comparison to pamidronate. In a pivotal double-blind randomized trial of patients with breast cancer and bone metastases, researchers compared ZA at 4 or 8 mg IV to pamidronate at 90 mg IV with no significant differences emerging between these agents in terms of the number of SREs or time to first SRE [86]. ZA is also the only BP approved for the prevention of SREs in mCRPC. Clodronate or pamidronate were not effective in improving the number of SREs in these patients [87,88,89,90]. In the ZA 039 trial of mCRPC, 643 patients were randomly assigned to the treatment with ZA at 8 or 4 mg IV or placebo q4w showing a significant reduction in the rate of SREs (49% vs. 38%, *p* = 0.0029) and an increase in the median time to first SRE in favor of ZA at 4 mg (the 8 mg arm was discontinued and patients reassigned to the 4 mg due to renal toxicity) [68,91]. ZA at a 4 mg IV dose q4w was also studied in the CALGB 90202 trial, in the castration-sensitive setting for advanced prostate cancer with bone metastasis. The study was terminated prematurely (645 of 680 planned accruals), because of the withdrawal of sponsor support. The median time to first SRE was 31.9 months in the ZA group vs. 29.8 with placebo (hazard ratio (HR) 0.97; *p* = 0.39), therefore not supporting the use of ZA in this setting [92].

Finally, ZA was further compared with ibandronate. In the ZICE trial, a non-inferiority study, ibandronate at the dose of 50 mg/daily was compared to ZA at the dose of 4 mg every 3 to 4 weeks in patients with breast cancer and bone metastasis [79]. Ibandronate was not non-inferior, with an annual rate of SREs of 0.499 (95% CI 0.454 to 0.549) for ibandronate and 0.435 (0.393–0.480) for ZA. However, given its safety profile and the fact that it is the only oral available BP some consider it as an alternative agent for unfit patients for which frequent hospital visits are not feasible [93].

Data from clinical trials with BPs in other solid tumors different than breast and prostate cancer are scarcer. ZA was evaluated in a placebo-controlled trial of 773 patients with skeletal metastases from cancers other than breast and prostate. Patients were randomly assigned to IV ZA at 8 mg, 4 mg or placebo q3w with concomitant antineoplastic therapy. As in other trials, ZA at 8 mg was subsequently reduced to 4 mg due to renal adverse events. SRE incidence was reduced in both ZA groups (38% for 4 mg and 35% for 8/4 mg of ZA vs. 44% for placebo; *p* = 0.127 and *p* = 0.023 for 4-mg and 8/4-mg groups, respectively). Moreover, ZA increased time to first SRE in the 4 mg group (median 230 days vs. 163 days for placebo; *p* = 0.023) [77,94]. Based upon these results, the use of ZA is recommended in patients with advanced solid tumors with evidence of bone metastases.

Bone disease is also a typical feature of multiple myeloma (MM). As a matter of fact, it is the cancer presenting most frequently bone metastasis (in up to 90% of patients). Bone lesions in MM are purely osteolytic (low radiographic density) with up to 60% of patients developing pathologic fractures over the course of their disease [95]. The efficacy of IV pamidronate in this setting was demonstrated in a clinical trial. Patients with stage III MM and at least one lytic lesion received either placebo or pamidronate 90 mg IV. The proportion of patients who developed any SRE was lower in the pamidronate group (28% vs. 44% *p* = 0.015) and the mean number of SREs per year was inferior in the pamidronate group (1.3) than in placebo-treated patients (2.2; *p* = 0.008) [96]. Similarly to breast cancer, ZA and pamidronate reduced SREs in a comparable fashion to those seen in MM patients [11].

In addition to BPs, osteoclast inhibition can also be achieved by targeting RANKL with denosumab, a fully humanized monoclonal antibody that binds to RANK and has demonstrated superiority to ZA in patients with bone metastatic disease from breast and prostate cancer. Side to ZA, denosumab is considered by international medical associations as an alternative treatment for patients with solid tumors and bone metastases [97]. Despite this equal indication; denosumab has demonstrated superiority to ZA in terms of time to first and subsequent SREs in patients with bone metastatic disease from breast and prostate cancer.

ZA was compared to denosumab for SRE reduction in three double-blind phase 3 studies, comparing ZA 4 mg IV q4w vs. denosumab 120 mg q4w. In Trial 20050103, which included 1904 patients with mCRPC, denosumab delayed the time to first SRE by 18% (20.7 vs. 17.1 months, HR 0.82, *p* = 0.0002) [74]. In Trial 20050136, 2046 patients with metastatic breast cancer involving the bone, denosumab delayed the time to first SRE compared to ZA (32.4 vs. 26.4 months HR 0.82, *p* = 0.01) [73]. Denosumab has also been studied in a variety of other malignancies in Trial 20050244 that compared denosumab and ZA in 1776 patients with MM or bone metastases from a solid tumor other than breast or prostate cancer. In this study denosumab was non-inferior to ZA in delaying time to first SRE (20.6 vs. 16.3 months, HR 0.84; 95%, *p* = 0.0007), but not statistically superior to ZA in delaying time to first SRE (*p* = 0.06) [75]. 

### 3.2. Bone-Targeted Radiopharmaceuticals: Radium-223

In the Alpharadin in Symptomatic Prostate Cancer trial (ALSYMPCA), 921 patients with mCRPC and symptomatic bone metastasis, were randomly assigned to Ra-223 at a dose of 50 kBq/kg IV dose q4w for six cycles or to placebo. Visceral involvement was not allowed (lung or liver), and patients could not have lymph nodes larger than 3 cm. Patients had to be previously treated with docetaxel, unless they were ineligible or refused to receive it. The primary endpoint was median overall survival (OS), which was improved in 3.6 months (14.9 vs. 11.3 months, HR 0.70, *p* < 0.001). Additional benefit was also shown for secondary endpoints, as time to first SSE that improved significantly (15.6 vs. 9.8 months, HR 0.66, *p* < 0,001). Likewise, a decrease in alkaline phosphatase > 30% occurred in 47% vs. 3% in the placebo group (*p* < 0.001) [80]. In this trial 41% of the patients were treated simultaneously with osteoclast-targeted agents (ZA exclusively). Of note, Ra-223 was associated with a survival advantage regardless of the use of BP. An additional advantage in delaying SSE was found for patients receiving ZA (19.6 vs. 10.2 months, HR 0.49, *p* = 0.00048). Patients not receiving ZA had a trend towards improved time to first SSE (11.8 vs. 8.4 months; HR 0.77, *p* = 0.07). Given these findings, there is no rationale for discontinuation of ZA when starting Ra-223 [98]. The safety, tolerability and efficacy profile of the combination of denosumab with Ra-223 is still unknown given that denosumab was not a standard during ALSYMPCA; however, there is also no clear rationale to advise against its use either.

### 3.3. Other Bone-Targeted Radiopharmaceuticals

While the only bone-targeted radiopharmaceutical to show improved survival in prostate cancer was Ra-223, others have demonstrated to improve pain control [99]. These include the beta emitters’ rhenium-186, rhenium-188, samarium-153 and strontium-89. Samarium-153 and strontium-89 were the most frequently used before approval of Ra-223. The use of these agents is mainly limited by the quality of the studies showing their efficacy and their myelosuppressive effects [99,100]. 

### 3.4. Adjuvant Use of Bone Targeted Agents

Several studies and a recent meta-analysis showed that BTAs are useful drugs to reduce the risk of breast cancer recurrence in postmenopausal women; its role in other tumors, namely prostate cancer, is less clear (Table 2).

Beyond the antiresorptive effects of BPs that reduce SREs and treatment related bone loss, pre-clinical evidence further suggests an anti-cancer activity for BPs [101]. In specific, BPs may act directly as cytotoxics, or indirectly by inhibiting angiogenesis, inhibiting the recruitment of tumor associated macrophages (TAMs), stimulating γδ T cells, or by acting synergistically with other chemotherapeutics. Finally, both BPs and denosumab disrupt the “vicious cycle” thus limiting tumor cells access to growth factors entrapped in the bone matrix. However, despite the strong preclinical rational for the anti-cancer action of BPs and denosumab, clinical studies performed in the metastatic setting never demonstrated a survival benefit. Furthermore, subsequent individual studies performed in the adjuvant setting intending to improve recurrence and survival and integrating BTAs in the conventional adjuvant regimen were inconsistent whether or not BTAs are useful drugs, especially for cancers other than breast. A recent meta-analysis of breast cancer studies added some clarity. Selected individual studies and the referred meta-analysis are detailed bellow, first for breast cancer and after for prostate cancer.

For breast cancer, the ABCSG-12 was a seminal phase III study showing a reduction in the risk of recurrence for patients treated with ZA [102,103]. In this trial, 1803 premenopausal women with endocrine-responsive early breast cancer were treated with goserelin plus tamoxifen or aromatase inhibitors with or without ZA (4 mg every six months for three years). Those treated with ZA had a 23% reduction in the risk of disease recurrence (absolute reduction of 3.4%; hazard ratio (HR) 0.77 (95% CI 0.60–0.99), *p* = 0.042) and a strong trend towards improved survival (HR 0.66 (95% CI 0.43–1.02), *p* = 0.064). An even more pronounced effect was noted for patients 40 years or older. 

The AZURE trial was a subsequent study intending to definitely solve this question. In this phase III randomized trial that included 3360 patients with axillary lymph-node metastasis or a T3–T4 primary tumor irrespective of menopausal status, after 59 months of median follow-up, those receiving ZA had an unimpressive 2% improvement in the relative risk of recurrence (DFS; HR 0.98 (95% CI 0.85–1.13), *p* = 0.79) and a non-significant 15% reduction in the risk of death (OS; HR 0.85 (95% CI 0.72–1.01), *p* = 0.07). Of note, in a planned sub-group analysis, late postmenopausal women (>5 years) had a remarkable benefit from ZA: a relative 25% reduction in the risk of recurrence (DFS) and 26% in the risk of death (OS). The fact that all patients in the ABCGS-12 were functionally postmenopausal (under ovarian suppression treatment) seems to reconcile the results of ABCSG-12 and AZURE trials. 

More recently, a large Early Breast Cancer Trialists’ Collaborative Group (EBCTCG) patient-level meta-analysis of trials of adjuvant BPs (amino and non-amino BPs) including 18,776 women with breast cancer showed a 18% reduction in the risk of death from breast cancer in the subset of postmenopausal women (HR 0.82 (95% CI 0.73–0.93), *p* = 0.002) [9]. This effect seems to mainly stem from the reduction of 28% in the risk bone recurrence (HR 0.72 (95% CI 0.60–0.86), *p* = 0.0002) and not from extra-osseous recurrences, namely loco-regional or visceral. Admitting the usefulness of adjuvant BPs, SWOG0307 trial is comparing three different agents: ZA, clodronate and ibandronate. Preliminary results presented at the American Society of Clinical Oncology annual meeting 2015 suggest no difference between these agents in terms of DFS or grade 3/4 adverse events [104]. However, a numerical difference in rate of osteonecrosis of the jaw was noted: highest for ZA (1.2%), then ibandronate (0.6%) followed by clodronate (0.3%). These data are consistent with the EBCTCG meta-analysis.

Denosumab is also being actively studied as an adjuvant treatment in breast cancer. Early data from the ABCSG-18 study, a phase III trial of 3425 postmenopausal patients with early hormone receptor positive BC receiving aromatase inhibitors treatment with or without denosumab (60 mg every six months), showed a reduction in the risk of recurrence of approximately 18% (HR 0.816 (95% CI 0.66–1.00), *p* = 0.051) [105]. The role of denosumab to improve cancer outcomes in the adjuvant setting (specifically bone related outcomes) is being prospectively tested in the D-CARE (NCT01077154) trial. Primary outcome is bone metastasis-free survival and estimated completion date of November 2017.

As for prostate cancer, adjuvant BTAs have a less established role. In the pivotal study ZEUS (Zometa European Study), a phase III trial that recruited 1433 patients with high-risk non-metastatic prostate cancer to be treated with or without every three months ZA, no difference was found in the proportion of patients developing bone metastases (17.1% vs. 17.0% without ZA; *p* = 0.95) [107]. In the RADAR study, a phase III trial recruiting 1071 men with locally advanced prostate cancer for the treatment with radiotherapy plus 6 or 18 month of androgen deprivation therapy (ADT) with or without 18 months of ZA, no differences were found in OS of prostate cancer specific survival between groups [108]. However, those patients with high Gleason score (i.e., 8–10) and treated with ADT for 18 months derived a benefit from ZA (in the form of the reduction of PSA progression and need for secondary therapeutic intervention). Of concern was the increased proportion of patients with bone progression in the subgroup treated for six months with ADT plus ZA vs. ADT only. These subgroup analyses should be interpreted with caution. Longer follow-up and other trials might add some extra clarity at these results before definitive conclusions can be taken.

If the improved cancer outcomes discussed above derive from any direct or indirect anti-cancer action of BTAs or rather derive from the altered “soil” resulting from BTAs action at the bone microenvironment is still not established; however, early data from breast cancer pointing to a shared effect between BPs and denosumab supports the latter. Moreover, it is intriguing that up to now only breast cancer patients seem to benefit from this treatment in terms of cancer outcomes, and unexpectedly not all, but only the subgroup of postmenopausal women. The fact that in prostate cancer, bone metastases are often blastic reflecting a strong interaction between cancers cells and osteoblasts could be a possible explanation for the lack of efficacy of BTAs that behave as anti-osteoclasts agents only to prevent disease relapse in bone. 

## 4. Future Directions and Conclusions

Bone metastasis is a rich field for research that has provided some practical therapeutic solutions. Targeting cancer cells continues to be the major task in the treatment of bone metastases but, because we understood the role of the microenvironment in cancer establishment in bone, we were able to explore further benefits from targeting osteoclasts too.

At present, we know that by inhibiting osteoclasts we can significantly decrease morbidity caused by bone metastases and, maybe even more interesting, we can prevent bone metastases after surgery of the primary tumor in postmenopausal women with breast cancer. With the known and approved BTAs we learned an effective way to target the host to treat cancer.

The understanding of the pathophysiology of bone metastases is still incomplete and by pursuing this task we may well find new treatments in bone metastases field. Some new forms of targeting osteoclasts and osteoblasts are object of clinical research (Table 3). Other possible targets, such as miRNAs, may gain clinical relevance in the future.

Hopefully, with the discovery of new therapies for patients with bone metastases we can develop new concepts that could be applicable to other sites of metastases. As we know that in many patients with breast cancer or prostate cancer bone is the only site of clinically detectable metastases for a considerable period of time these clinical facts should represent an opportunity to study not only the organotropic attraction of cancer cells to organs such as lung or liver, but also the pathophysiology of cancer progression in those organs.

## Figures and Tables

**Table 1:** Current data on the use of bone-targeted agents in patients with bone metastasis. Abbreviations: CI, confidence interval; HR, hazard ratio; NR, not reached; SC, subcutaneous; ZA, zoledronic acid; MM, multiple myeloma; EMA, European Medical Agency; FDA, Food and Drug Administration; SRE, skeletal related events; RANKL, receptor activator of NFκB ligand; BP, bisphosphonates; IV, intravenous; PO, *per os*.

Agent and Dose	Drug Class and Target	EMA Label	FDA Label	Time to First SRE (if Not Otherwise Specified)	Overall Survival	Refs.
Denosumab; 120 mg, SC, every 4 weeks	Fully human monoclonal antibody; Anti-RANKL	Label; Prevention of SREs (pathological fracture, radiation to bone, spinal cord compression or surgery to bone) in adults with bone metastases from solid tumors.	Label; Prevention of skeletal-related events in patients with bone metastases from solid tumors; excludes bone metastases from MM.	Breast (vs. ZA): NR vs. 26.4 months; HR 0.82 (95% CI, 0.71 to 0.95), *p* = 0.01;	Overall (vs. ZA): No benefit.	[8,73,74,75]
				Prostate (vs. ZA): 20.7 vs. 17.1 months; HR 0.82 (95% CI 0.71–0.95), *p* < 0.001;		
				Other solid tumors and MM (vs. ZA): 20.6 vs. 16.3 months; HR 0.84 (95% CI 0.71–0.98), *p* < 0.001;		
				Overall (vs. ZA): 27.7 vs. 19.5 months; HR 0.83 (95% CI 0.76–0.90), *p* < 0.001.		
ZA; 4 mg, IV, every 3 to 4 weeks	Amino-bisphosphonat; farnesyl diphosphate synthase inhibitor	Prevention of skeletal-related events (pathological fractures, spinal compression, radiation or surgery to bone, or tumor-induced hypercalcemia) in adult patients with advanced malignancies involving bone.	Patients with MM and patients with documented bone metastases from solid tumors, in conjunction with standard antineoplastic therapy. Prostate cancer should have progressed after treatment with at least one hormonal therapy.	Breast (vs. placebo): NR vs. 360 days; HR 0.56 (95% CI 0.36–0.87); *p* = 0.009	Overall (any BP vs. control): HR 1.01 (95% CI 0.92–1.11); *p* = 0.87; No benefit.	[68,76,77,78]
				Prostate (vs. placebo): 488 vs. 321 days; HR 0.68 (95% CI 0.51–0.91); *p* = 0.009		
				Lung and other solid tumors (vs. placebo): 230 vs. 155 days; HR 0.70 (95% CI NR); *p* = 0.006		
Ibandronic acid; 50 mg, PO, daily	Amino-bisphosphonat; farnesyl diphosphate synthase inhibitor	Prevention of skeletal events (pathological fractures, bone complications requiring radiotherapy or surgery) in patients with breast cancer and bone metastases.	Off-label	Breast (vs. ZA): annual rate ratio for SREs 1.148 (95% CI 0.967–1.362); did not demonstrate non-inferiority to ZA.	Overall (any BP vs. control): HR 1.01 (95% CI 0.92–1.11); *p* = 0.87; No benefit.	[79]
Ra-223; 50 kBq per kilogram, every 4 weeks for 6 administrations	Alpha-emitter; DNA damage	Treatment of adults with castration-resistant prostate cancer, symptomatic bone metastases and no known visceral metastases.	Treatment of patients with castration-resistant prostate cancer, symptomatic bone metastases and no known visceral metastatic disease	Prostate (vs. placebo): 15.6 months vs. 9.8 months; 0.66 (95% CI, 0.52–0.83); *p* < 0.001	Prostate (vs. placebo): 14.0 vs. 11.2 months; HR 0.70 (95% CI 0.58–0.83); *p* < 0.001	[80]

**Table 2:** Current data on the use of bone-targeted agents in the adjuvant treatment of breast and prostate cancers. ADT, androgen deprivation therapy; RT, radiotherapy.

Agent	Group of Patients; Number of pts	EMA/FDA Label	Bone Recurrence	Disease Recurrence	Cancer Mortality	Refs.
**Breast cancer**						
Bisphophonates	Overall; *n* = 18,766	Off-label	HR 0.83 (95% CI 0.73–0.94); *p* = 0.004	HR 0.94 (95% CI 0.87–1.01); *p* = 0.08	HR 0.91 (95% CI 0.83–0.99); *p* = 0.04	[9]
	Postmenopausal; *n* = 7388		HR 0.72 (95% CI 0.60–0.86); *p* = 0.0002	HR 0.86 (95% CI 0.78–0.94); *p* = 0.002	HR 0.82 (95% CI 0.73–0.93); *p* = 0.002	
Denosumab	Postmenopausal; *n* = 3425	Off-label	-	(any recurrence or death) HR 0.82 (95% CI 0.66–1.00); *p* = 0.0515	-	[105,106]
**Prostate cancer**						
Zoledronic acid	High risk disease; 1393	Off-label	14.7% vs. 13.2% in the control group; HR 1.075 (95% CI 0.81–1.44); *p* = 0.62	-	116 vs. 122 deaths in the control group; log-rank *p* = 0.76	[107]
	Locally advanced disease treated with RT and ADT ± ZA		See text.			[108]

**Table 3:** Phase III trials testing new bone targeted agents (ongoing or closed/completed; excluding bisphosphonates, denosumab and radium-223) for the treatment of bone metastasis. For an in-depth analysis of clinical trials testing bone-targeted agents, see reference [109].

Target	Agent	NCT	Combination Therapy/Comparator	Tumor Type	Bone-Specific Endpoints	Trial Status
Cathepesin K	Odanacatib	NCT00691899	None/Placebo	Prostate	Bone metastasis-free survival	Withdrawn
		NCT00692458	None/Placebo	Breast	Development of bone metastasis	Withdrawn
c-Src	Dasatinib	NCT00744497	Docetaxel, prednisone/Placebo	Prostate	Time to first SRE; reduction of NTX from baseline	Completed
Endothelines	Atrasentan	NCT00036543	None/Placebo	Prostate	None	Completed
		NCT00134056	Docetaxel, prednisone/placebo	Prostate	None	Ongoing
	Zibotentan	NCT00554229	None/Placebo	Prostate	Incidence of SRE; New bone metastases	Completed
mTOR	Everolimus	NCT00863655	Exemestane/Placebo	Breast	None	Completed
MET/VEGFR	Cabozantinib	NCT01605227	None/Prednisone	Prostate	Bone scan response	Completed
		NCT01522443	None/Mitoxantrone, prednisone	Prostate	Bone scan response	Terminated

NTX, N-terminal telopeptide; mTOR, mammalian target of rapamycin; MET/VEGFR, MET/Vascular endothelial growth factor.
